# Genome-wide investigation of WRKY gene family in pineapple: evolution and expression profiles during development and stress

**DOI:** 10.1186/s12864-018-4880-x

**Published:** 2018-06-25

**Authors:** Tao Xie, Chengjie Chen, Chuhao Li, Jiarou Liu, Chaoyang Liu, Yehua He

**Affiliations:** 1 Key Laboratory of Biology and Germplasm Enhancement of Horticultural Crops in South China, Ministry of Agriculture, South China Agricultural University, Guangzhou, 510642 China; 2 College of Horticulture, South China Agricultural University, Guangzhou, 510642 China

**Keywords:** Pineapple, WRKY, Genome-wide, Expression patterns, Abiotic stress

## Abstract

**Background:**

WRKY proteins comprise a large family of transcription factors that play important roles in many aspects of physiological processes and adaption to environment. However, little information was available about the *WRKY* genes in pineapple (*Ananas comosus*), an important tropical fruits. The recent release of the whole-genome sequence of pineapple allowed us to perform a genome-wide investigation into the organization and expression profiling of pineapple *WRKY* genes.

**Results:**

In the present study, 54 pineapple *WRKY* (AcWRKY) genes were identified and renamed on the basis of their respective chromosome distribution. According to their structural and phylogenetic features, the 54 *AcWRKYs* were further classified into three main groups with several subgroups. The segmental duplication events played a major role in the expansion of pineapple *WRKY* gene family. Synteny analysis and phylogenetic comparison of group III *WRKY* genes provided deep insight into the evolutionary characteristics of pineapple *WRKY* genes. Expression profiles derived from transcriptome data and real-time quantitative PCR analysis exhibited distinct expression patterns of *AcWRKY* genes in various tissues and in response to different abiotic stress and hormonal treatments.

**Conclusions:**

Fifty four *WRKY* genes were identified in pineapple and the structure of their encoded proteins, their evolutionary characteristics and expression patterns were examined in this study. This systematic analysis provided a foundation for further functional characterization of *WRKY* genes with an aim of pineapple crop improvement.

**Electronic supplementary material:**

The online version of this article (10.1186/s12864-018-4880-x) contains supplementary material, which is available to authorized users.

## Background

The WRKY transcription factors (TFs) are one of the largest families in higher plants and are found throughout the green lineage [1]. The most prominent feature of the WRKY proteins is the 60 amino acid long WRKY domain, which comprises the highly conserved signature WRKYGQK followed by a C2H2- or C2HC-type of zinc-finger motif. Both heptapetide sequence and zinc-finger motif are required for the high binding affinity of WRKY TFs to the consensus W-box cis-elements [2, 3]. Based on the number of WRKY domains and the structure of their zinc-finger motifs, the WRKY proteins can be classified into three main groups (I–III), those with two WRKY domains belong to Group I, while those with one WRKY domain belong to Group II or III [4].

Since the first WRKY gene, SPF1, was cloned from sweet potato [5], a large number of WRKY proteins have been experimentally identified from various plant species. Substantial evidence indicates that the WRKY transcription factors are involved in plant defense regulatory networks, including response to various biotic and abiotic stresses [6, 7]. The WRKY TFs are one of the best-characterized classes of plant defense transcription factors and at the forefront of research on plant defense responses. The WRKY proteins play a key role in plant defense against biotic stresses including bacterial, fungal and viral pathogens [8–10]. A large number of WRKY TFs are induced by abiotic stresses and take part in the regulation of plant tolerance to abiotic stress [11]. For example, VvWRKY11 from grapevine is involved in the response to dehydration stress [12]. AtWRKY75 is induced in the plant during phosphate deficiency while suppression of WRKY75 expression leads to the increased susceptibility to phosphate stress [13]. The WRKY proteins play central roles in various aspects of physiological processes, including senescence [14], seed dormancy and germination [15], embryogenesis [16], trichome development, modulation of flowering time [17, 18], fruit flavor [19], biosynthesis of secondary metabolites [20–22]. They are also key components in some signal transduction processes mediated by plant hormones such as abscisic acid (ABA) and salicylic acid (SA) [6]. The Arabidopsis WRKY39 positively regulates the cooperation between the SA- and JA-activated signaling pathways that mediate response to heat stress [23]. OsWRKY31 mediates crosstalk between auxin and the defense signal transductions [24].

The WRKY proteins are conserved in evolutional history throughout the plant kingdom, and the expansions of this family seem to be related to the evolution and diversity of the plants [25]. Although WRKY TFs are considered as plant-specific, they were also reported in non-plant species, e.g. protist, slime mold, and unicellular algae [4]. The evolution of the WRKY gene family provide insights into how biotic and abiotic stress responses and signaling evolved as plant went from simpler, unicellular to more complex, multicellular flowering plants. The availability of increasing numbers of sequenced genomes has facilitated the evolutionary studies of the WRKY genes family, and large-scale genome-wide analysis of WRKY genes have been described in many different species, which would be helpful to understanding their evolutionary origin and biological functions.

Pineapple, *Ananas comosus* (L.) Merr., is the only species of the Bromeliaceae family grown commercially for its fruit [26]. After banana and citrus, it is the third most important tropical fruit in world production, and is found in almost all the tropical and subtropical regions of the world [27]. The environmental stresses significantly affect the growth and development of the pineapple plant. The pineapple fruit can be injured by sunburn under high temperature, the low temperature can result in diminished growth and the plant is usually severely damaged by frost [26]. To a certain extent the pineapple can survive under drought conditions owing to the plant morphological features and its crassulacean acid metabolism. Nevertheless, prolonged extreme droughts affect growth and yield dramatically. The biotic stresses like pests, diseases, and weeds can also cause significant yield loss in plantation pineapple production. Additionally, pineapple is also an important species in the research of the monocot evolution owing to its pivotal phylogenetic position at the base of the order Poales [28]. The pineapple is cultivated worldwide and has great economic and research value and so there is considerable interest in identifying important functional genes.

The WRKY gene family has been extensively studied in many plant species. However, current basic knowledge of WRKY proteins in pineapple is still limited. Due to the importance of the WRKY genes in various physiological programs, it would be of interest to make a systematic investigation of the WRKY family in pineapple. Recent completion of the pineapple genome sequencing provided an opportunity to reveal the organization, expression and evolutionary traits of pineapple WRKY gene family at the genome-wide level [29]. In the present study, we identified 54 pineapple *WRKY* genes and classified them into three main groups. The comprehensive analysis including the exon-intron organization, motif compositions, gene duplications, chromosome distribution, phylogenetic and synteny analysis were further investigated. Global expression analysis was performed to identify involvement of specific WRKY gene family members in different biological processes in pineapple. This study provided valuable clues for functional characterization of WRKY gene family members in pineapple.

## Results

### Identification of the WRKY proteins in pineapple

A total of 56 candidate gene models corresponding to the Pfam WRKY family were originally obtained. The annotation of these gene models were further checked using available pineapple transcriptome data. Ten erroneously predicted WRKY gene models were manually curated and two redundant sequences (*Aco030791.1* and *Aco025780.1*) were then removed. Finally, 54 gene models were selected and annotated as being pineapple *WRKY* genes based on the presence of apparently complete WRKY domains. The validated *AcWRKY* gene sequences were available in Additional file 1. A total of 51 *WRKY* genes could be mapped on the linkage groups and were renamed from AcWRKY1 to AcWRKY51 based on their order on the linkage groups. Three *WRKY* genes (*Aco027950.1*, *Aco028684.1*, and *Aco031477.1*) that could not be conclusively mapped to any linkage groups were renamed AcWRKY52- AcWRKY54 respectively.

Gene characteristics, including the length of the CDS (Coding Sequence), the length of the protein sequence, the protein molecular weight (MW), isoelectric point (pI), and the subcellular localization were analyzed (Additional file 1). Among the 54 AcWRKY proteins, AcWRKY14 was identified to be the smallest protein with 122 amino acid (aa), whereas the largest one was AcWRKY23 (1320 aa). The MW of the proteins ranged from 13.7 to 144.5 kDa, and the pI ranged from 5.11 (AcWRKY10) to 10.08 (AcWRKY54). The predicted subcellular localization results showed that 51 AcWRKY proteins were located in the nuclear region, whereas three proteins were located in the chloroplast.

### Multiple sequence alignment, phylogenetic analysis, and classification of *AcWRKY* genes

**Fig. 1:**
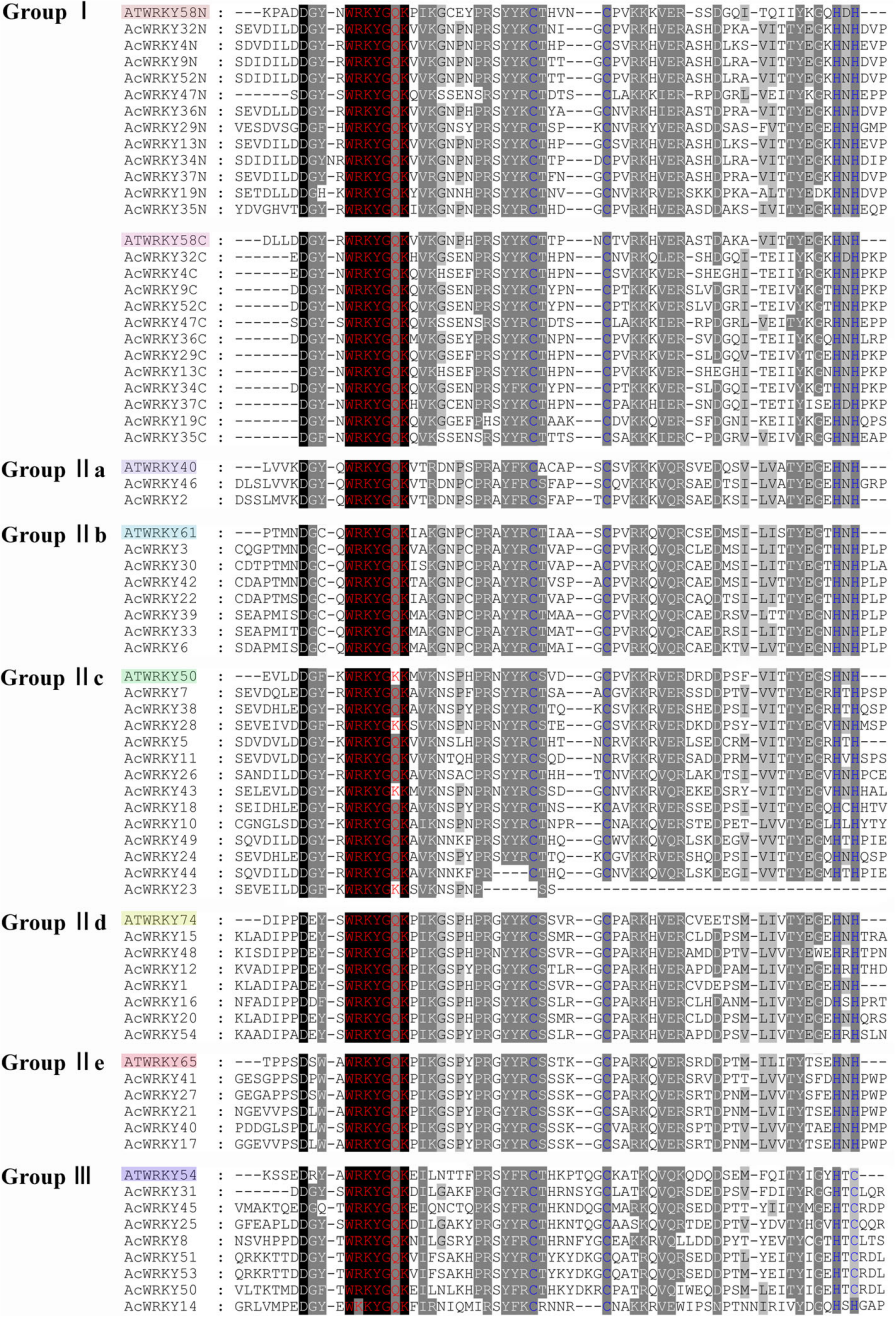
Alignment of multiple AcWRKY and selected AtWRKY domain amino acid sequences. ‘N’ and ‘C’ indicate the N-terminal and C-terminal WRKY domain of a specific WRKY protein

The phylogenetic relationship of the AcWRKY proteins was examined by multiple sequence alignment of their WRKY domains, which span approximately 60 amino acids. The WRKY domain of seven different Arabidopsis WRKY proteins (ATWRKY58, 40, 61, 50, 74, 65, 54) from each of the groups or subgroups, were randomly selected as representatives for the further comparison. As shown in Fig. 1, the sequences in the WRKY domain were highly conserved. A total of 50 AcWRKY proteins were found to have the highly conserved sequence WRKYGQK, while the others (AcWRKY14, AcWRKY23, AcWRKY27 and AcWRKY43) vary by a single amino acid.

**Fig. 2:**
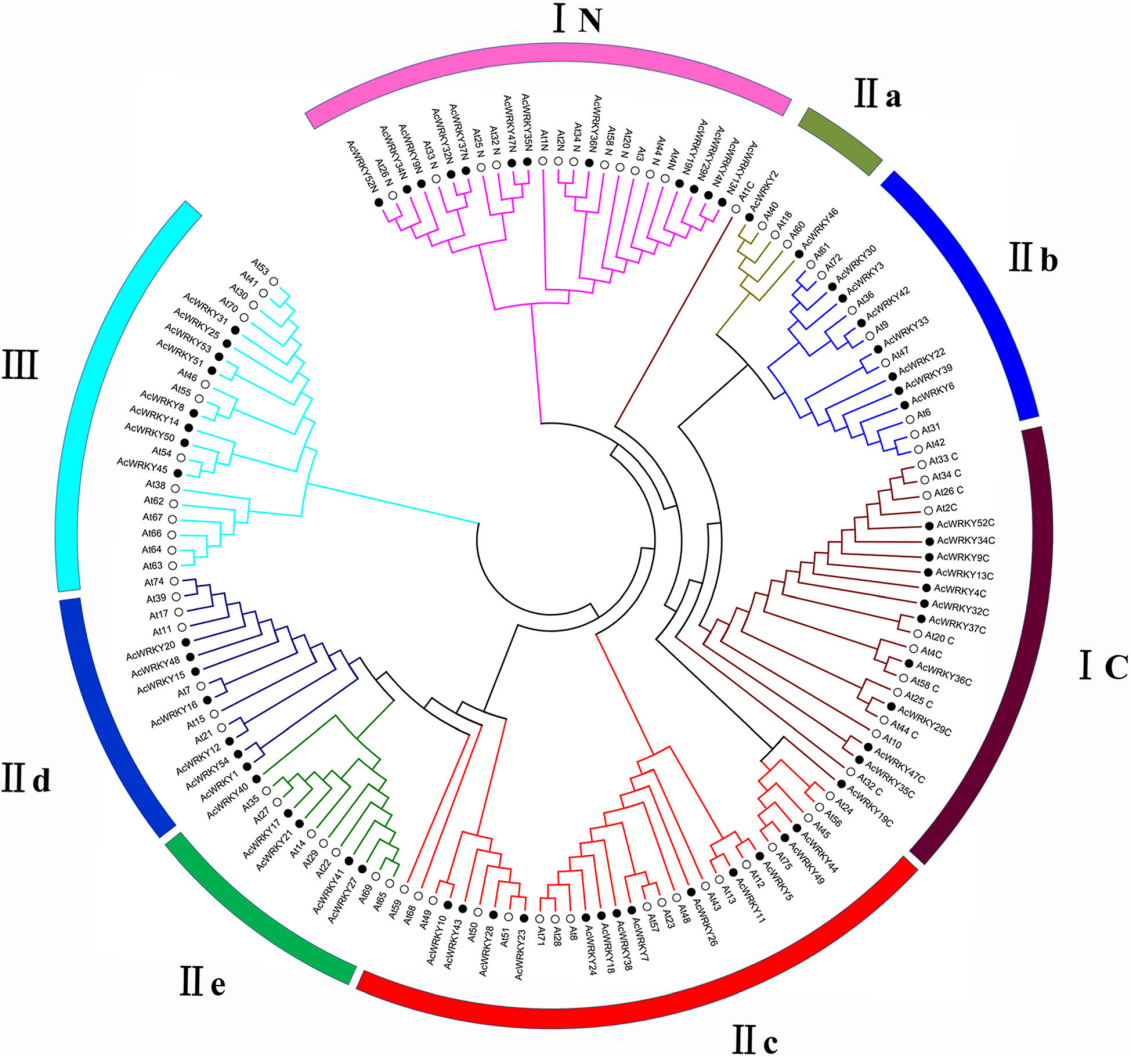
Unrooted phylogenetic tree representing relationships among WRKY domains of pineapple and Arabidopsis. The different-colored arcs indicate different groups (or subgroups) of WRKY domains. Group I proteins with the suffix ‘N’ or ‘C’ indicates the N-terminal or the C-terminal WRKY domains. The black solid circles and hollow circles represent WRKY domain from pineapple and Arabidopsis, respectively. WRKY proteins from Arabidopsis with the prefix ‘At’ indicate ‘AtWRKY’

The phylogenetic analysis (Fig. 2) indicated that the pineapple WRKY domains could be divided into three large groups corresponding to group I, II and III in Arabidopsis as defined by Eulgem et al. [2]. Among the 54 AcWRKY proteins, 12 belong to group I, 34 to group II, and 8 to group III. The 14 members from group I all contained two WRKY domains and C2H2-type zinc-finger motifs (C-X_4_-C-X_22–23_-H-X-H), without the domain loss events which usually happened in some monocotyledonous species [30]. The N-terminal and C-terminal WRKY domains were clustered in different clades, which may reflect the parallel evolution of the two domains. The WRKY members in group II can be further clustered into five subgroups (IIa-IIe), two WRKY proteins belong to IIa, 7 to IIb, **13** to IIc, 7 to IId, and 5 to IIe. Seven of the eight members in group III contain the C2HC-type zinc fingers (C-X_7−_C-X_23_-H-X-C), whereas the remaining AcWRKY14 possess a C2H2 type of zinc finger motif. The extended WRKY domains, which were found in several group III WRKY proteins in some monocot species like rice and Brachypodium [30], were not observed in pineapple.

The ‘leucine-rich repeat’ (LRR) motif, typical domain of resistance (R) proteins, was detected in AcWRKY23 belonging to group IIc. AcWRKY23 could be further characterized as an R protein-WRKY gene in pineapple. The existence of such chimeric proteins was one of the unusual features of the WRKY gene family in flowering plants like Arabidopsis and rice [25]. Further phylogenetic and the protein architecture analysis for AcWRKY23 and several R protein-WRKY proteins in other species (Additional file 2) indicated that AcWRKY23 was not belong to any group that have been characterized in Rinerson et al. [25].

### Gene structure and motif composition of pineapple WRKY gene family

**Fig. 3:**
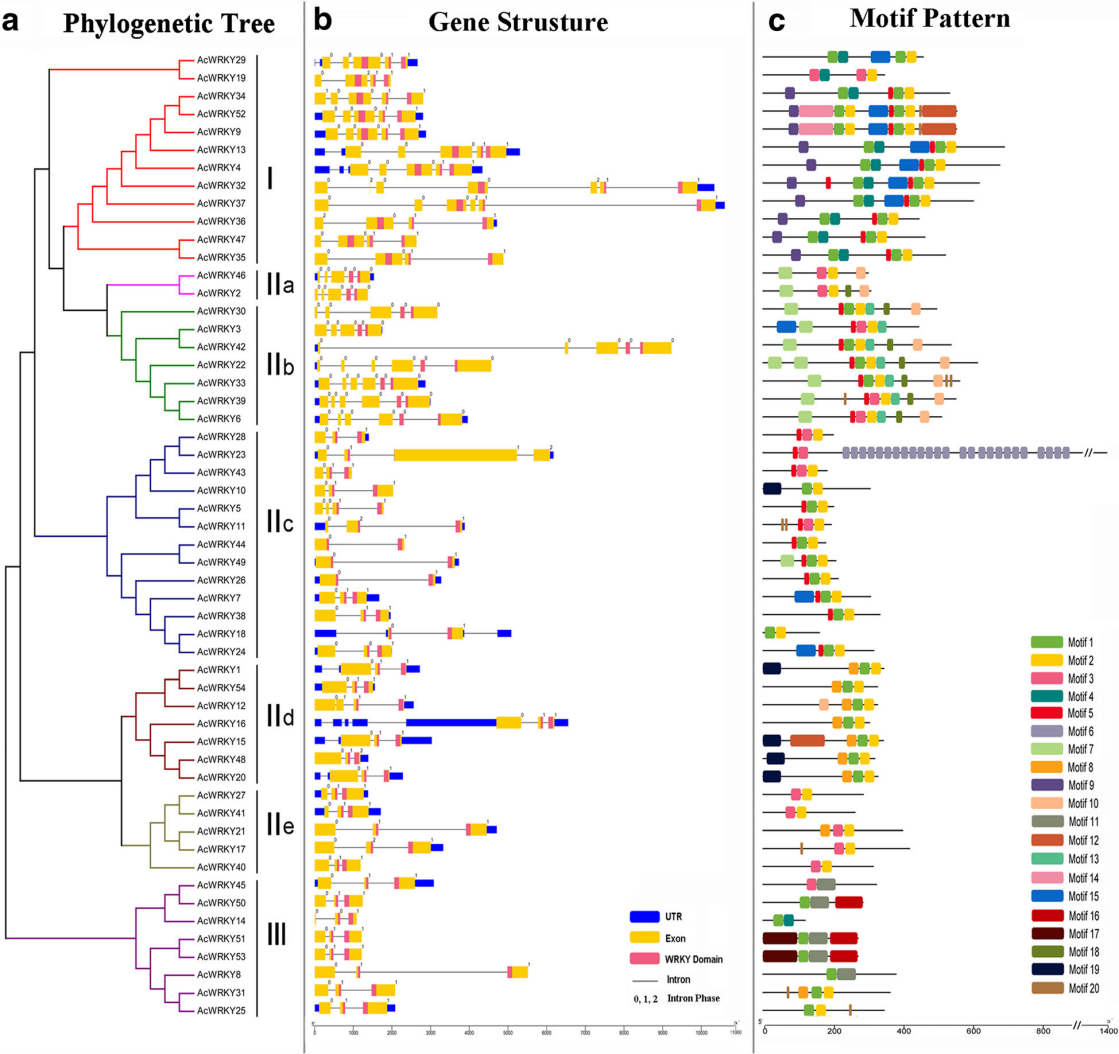
Phylogenetic relationships, gene structure and architecture of conserved protein motifs in WRKY genes from pineapple. **a** The phylogenetic tree was constructed based on the full-length sequences of pineapple WRKY proteins using MEGA 5 software. Details of clusters are shown in different colors. **b** Exon-intron structure of pineapple WRKY genes. Blue boxes indicate untranslated 5′- and 3′-regions; yellow boxes indicate exons; black lines indicate introns. The WRKY domains are highlighted by red boxes. The number indicates the phases of corresponding introns. **c** The motif composition of pineapple WRKY proteins. The motifs, numbers 1–20, are displayed in different colored boxes. The sequence information for each motif is provided in Additional file 2. The length of protein can be estimated using the scale at the bottom

The exon-intron organizations of all the identified *AcWRKY* genes were examined to gain more insight into the evolution of the WRKY family in pineapple. As shown in Fig. 3b, all *AcWRKY* genes possessed two to six exons (four with two exons, 27 with three exons, six with four exons, 11 with five exons, and six with six exons). Genes with only one exon were not observed. Genes within the same group usually have a similar structure, for example, all group IIe members contained three exons and two introns. Further analyses indicated that all *AcWRKY* genes contained an intron in their respective WRKY domains. The distribution of introns and the intron phase were coincident with the alignment clusters of *AcWRKY* genes. The V-type intron, a phase-0 intron, was only observed in group IIa and IIb. The R-type intron (a phase-1 intron) was widely distributed in all the other groups (group I, IIc, IId, IIe and III), similar with that in rice and Arabidopsis [25]. No introns were found in the N-terminal WRKY domains of genes belonging to group I.

A schematic representing the structure of all AcWRKY proteins was constructed from the MEME motif analysis results. As exhibited in Fig. 3c, other than motifs 1 and 2 which are the WRKY domains widely distributed, AcWRKY members within the same groups were usually found to share a similar motif composition (Additional file 3). For example, motif 9 is unique to group I, whereas motif 10 is specific to group IIa and IIb. The clustered AcWRKY pairs, i.e. AcWRKY47/35, AcWRKY51/53, showed highly similar motif distribution. The similar motif arrangements among AcWRKY proteins within subgroups indicated that the protein architecture is conserved within a specific subfamily. The functions of most of these conserved motifs remain to be elucidated. Overall, the conserved motif compositions and similar gene structures of the WRKY members in the same group, together with the phylogenetic analysis results, could strongly support the reliability of the group classifications.

### Evolutionary analysis of group III *WRKY* genes in pineapple and several different species

The group III WRKY genes are thought to have originated after the divergence of the monocots and dicots, and seem to have played a key role in plant adaption and evolution [31]. Here, we further investigated the duplication and diversification of group III genes during evolution, basing on the available pineapple WRKY III genes. A phylogenetic tree of WRKY III complete protein sequences from ten representative species, including six monocots (pineapple, rice, maize, banana, Brachypodium and millet) and four dicots (Arabidopsis, grape, tomato and poplar), was constructed using MEGA 5.0 [30–35].

**Fig. 4:**
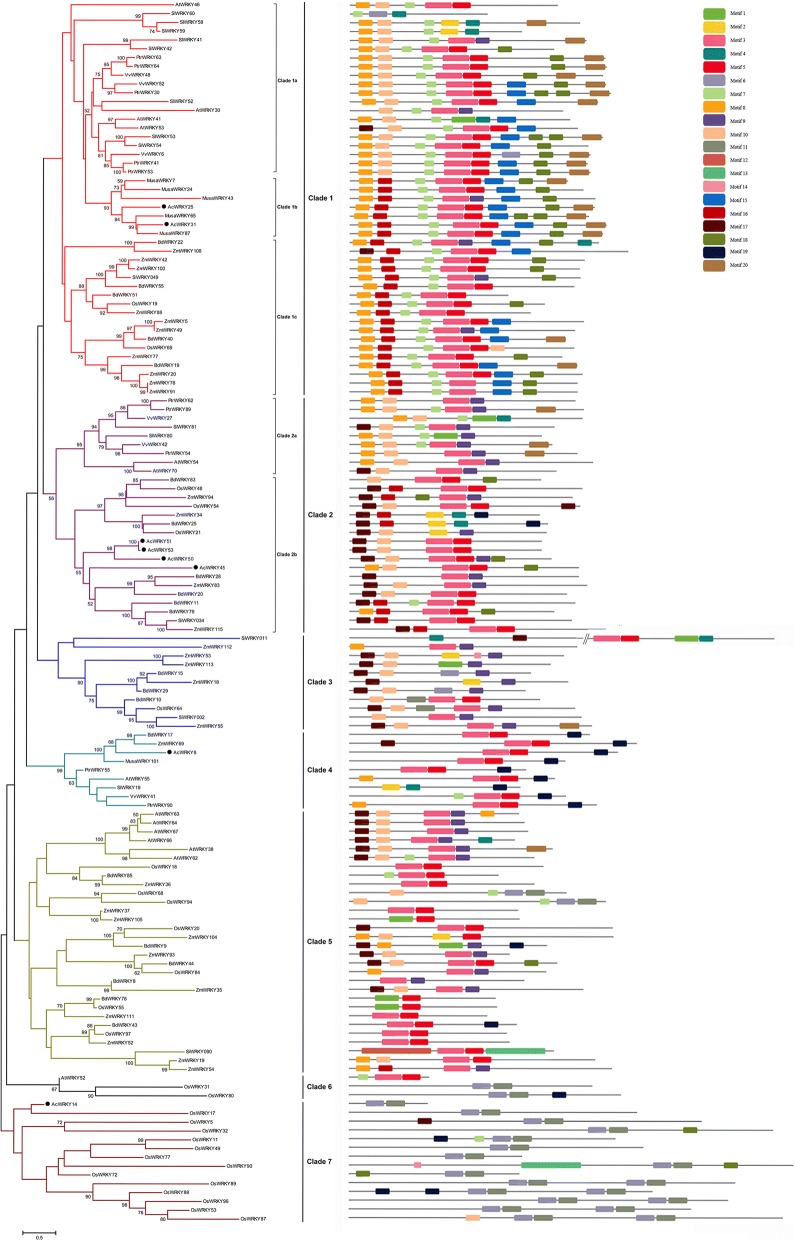
Phylogenetic relationships and motif compositions of group III WRKY proteins from ten different plant species. Left panel: an unrooted phylogenetic tree constructed using MEGA 5.0 by the Neighbor-Joining method. The proteins are clustered into seven main clades with several subclades. Subtrees branch lines are colored indicating different clades. The black solid circles indicate group III WRKY proteins from pineapple. Right panel: distribution of conserved motifs in the group III WRKY proteins. The different-colored boxes represent different motifs and their position in each WRKY protein sequence

As indicated in Fig. 4, the WRKY III proteins were divided in to seven clades by the phylogenetic tree. Each of the ten species contributed at least one WRKY III gene to Clade 1 and Clade 2, and these two clades were further divided into several different subclades. WRKY members from the phylogenetically closer species were clustered together within the two clades. For example, Clade 1a contained member from dicots, Clade 1b possessed member from pineapple and banana, the non-grass monocots, whereas Clade 1c contained proteins only from the grass species. Additionally, Clade 3 contained the grass-species specific WRKY members. Nine WRKY genes from eight different species were included in Clade 4, indicating that they might be orthologues that originated from a single ancestral gene. Interestingly, AcWRKY14 was clustered together with a series of rice WRKY III proteins (in Clade 7), implying that the different evolutionary patterns of group III WRKY in rice and pineapple may occur after their divergence.

We also use the MEME web server to search the conserved motifs which were shared with the WRKY III proteins. A total of 20 distinct conserved motifs were found, motif 1, motif 2, motif 3 and motif 6 were found to encode the WRKY domain. As illustrated in Fig. 4, most WRKY members within the same clade, especially the most closely related members, usually shared common motif compositions(e.g. AcWRKY8 and MusaWRKY101), indicating potential functional similarities among WRKY proteins. Motif 15 was unique to the members in Clade 1, which may be important to the functions of unique WRKY III protein. Interestingly, motif 16 is only observed in the WRKY III proteins from monocots. Motif 6 and adjacent motif 11 were only co-existed in AcWRKY14 and several rice WRKY III proteins (in Clade5, 6 and 7). These specific motifs may contribute to the functional divergence of WRKY genes.

### Chromosomal distribution and synteny analysis of *AcWRKY* genes

**Fig. 5:**
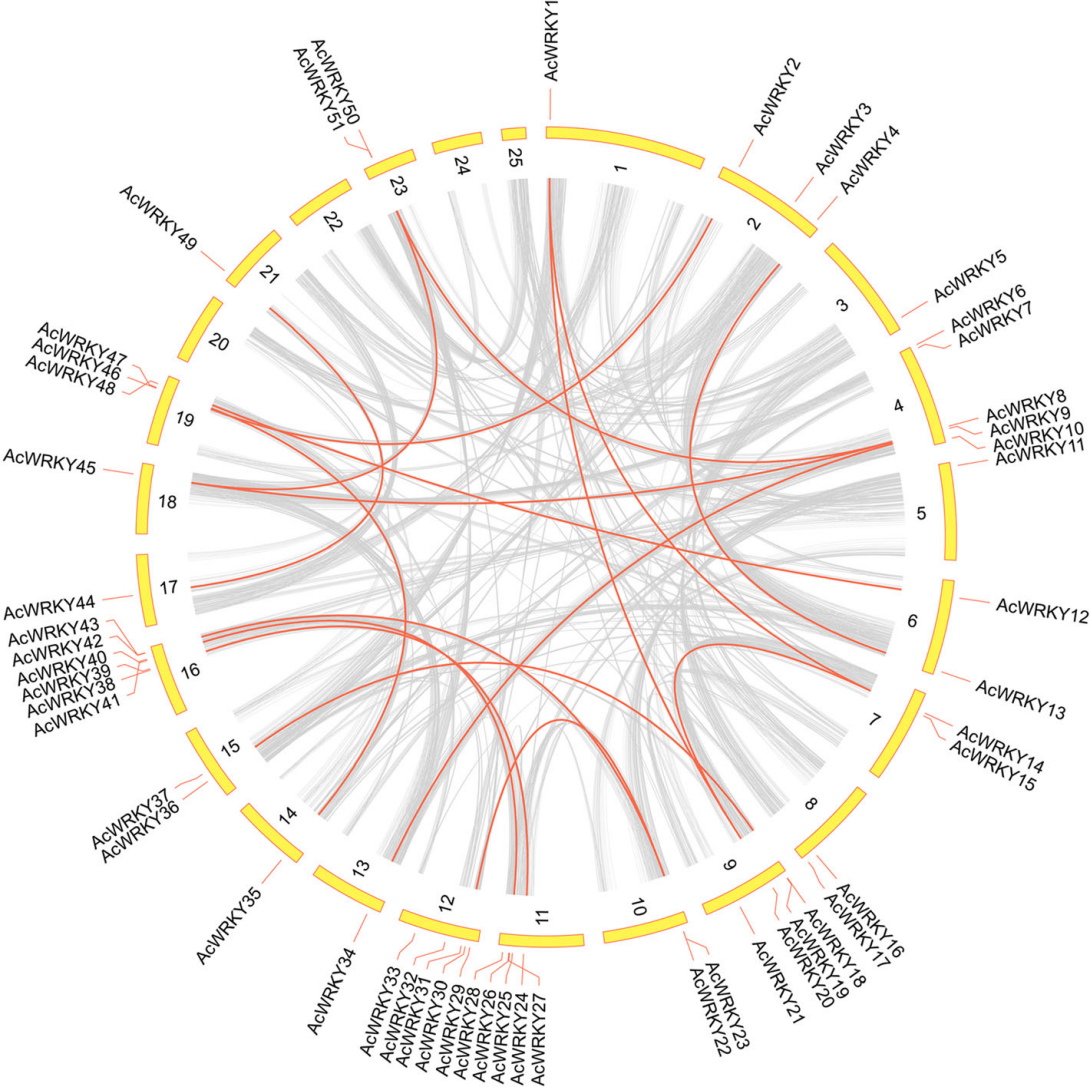
Schematic representations for the chromosomal distribution and interchromosomal relationships of pineapple WRKY genes. Gray lines indicate all synteny blocks in the pineapple genome, and the red lines indicate duplicated WRKY gene pairs. The chromosome number is indicated at the bottom of each chromosome

Figure 5 showed that the *AcWRKY* genes were unevenly distributed on the 25 pineapple linkage groups (LG) except LG 20, 22 and 25. LG16 contained the largest number of WRKY genes (6). Some linkage groups (e.g. LG16, LG12) have more genes, whereas others have few; some LGs have only one gene (e.g. LG03). There were no positive correlation between the LG length and the number of WRKY genes.

According to the descriptions of Holub [36], a chromosomal region within 200 kb containing two or more genes is defined as a tandem duplication event. Fourteen *AcWRKY* genes (*AcWRKY18/19*, *AcWRKY22/23*, *AcWRKY26/27*, *AcWRKY38/39*, *AcWRKY42/43*, *AcWRKY46/47*, and *AcWRKY50/51*) were clustered into seven tandem duplication event regions on pineapple linkage group 09, 10, 11, 16, 19, and 23. LG16 had two clusters, indicating a hot spot of WRKY gene distribution. Besides the tandem duplication events, 17 segmental duplication events with 27 WRKY genes were also identified with BLASTP and MCScanX methods (Additional file 4). These results indicated that some *AcWRKY* genes were possibly generated by gene duplication and the segmental duplication events played a major driving force for AcWRKY evolution.

**Fig. 6:**
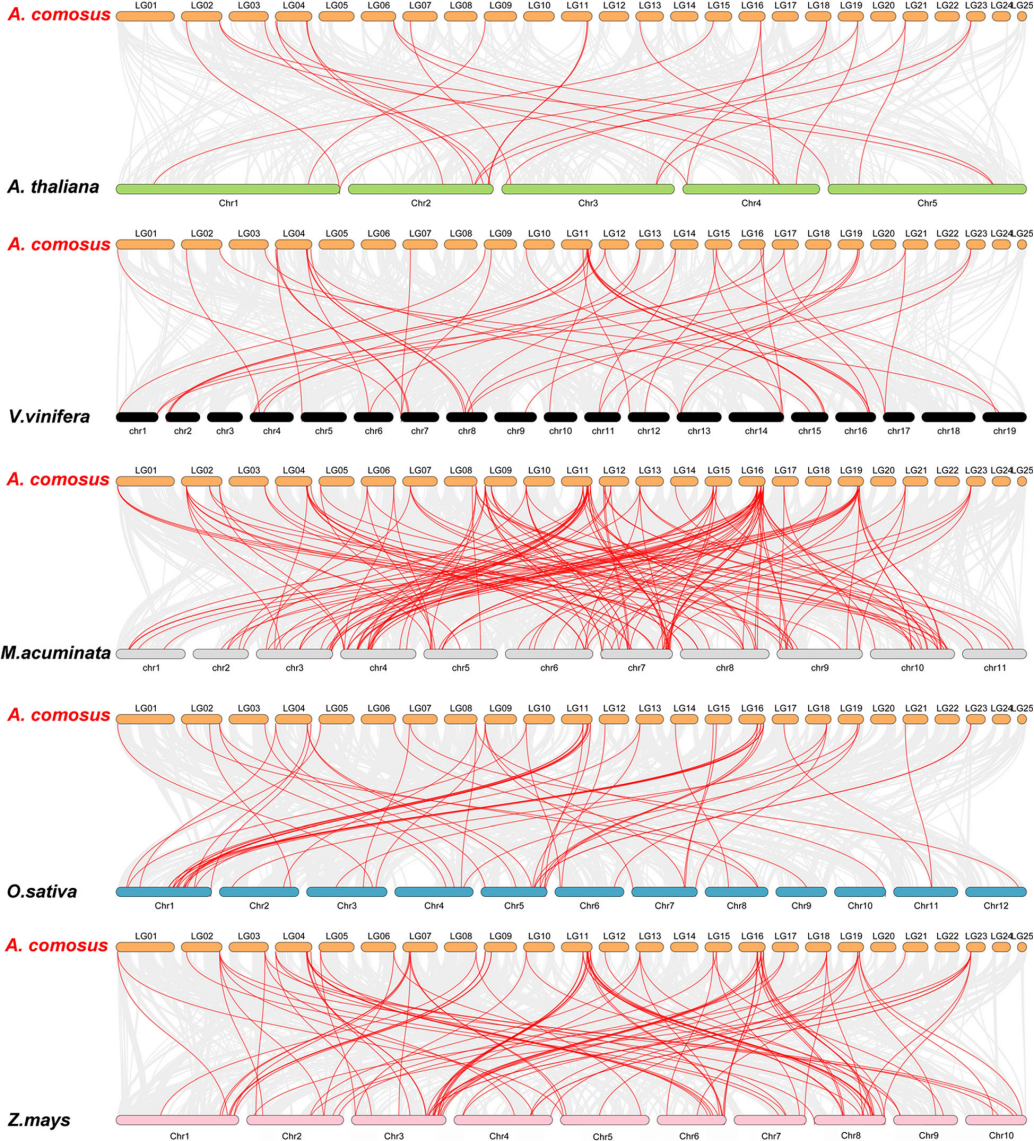
Synteny analysis of WRKY genes between pineapple and five representative plant species. Gray lines in the background indicate the collinear blocks within pineapple and other plant genomes, while the red lines highlight the syntenic WRKY gene pairs. The specie names with the prefixes ‘*A. comosus*’, ‘*A. thaliana*’, ‘*V. vinifera*’, ‘*M. acuminate*’, ‘*O. sativa*’ and ‘*Z. mays*’ indicate *Ananas comosus*, *Arabidopsis thaliana*, *Vitis vinifera*, *Musa acuminate*, *Oryza sativa*, and *Zea mays*, respectively

To further infer the phylogenetic mechanisms of pineapple WRKY family, we constructed five comparative syntenic maps of pineapple associated with five representative species, including two dicots (Arabidopsis and grape) and three monocots (banana, rice and maize) (Fig. 6). A total of 44 *AcWRKY* genes showed syntenic relationship with those in banana, followed by maize (39), rice (37), grape (33) and Arabidopsis (18) (Additional file 5). The numbers of orthologous pairs between the other five species (bananas, maize, rice, grape and Arabidopsis) were 145, 89, 56, 48 and 25. Some *AcWRKY* genes were found to be associated with at least three syntenic gene pairs (particularly between pineapple and bananas WRKY genes), such as *AcWRKY36* and *AcWRKY40*, guessed that these genes may have played an important role of WRKY gene family during evolution. Significantly, some WRKY collinear gene pairs identified between pineapple and rice were anchored to the highly conserved syntenic blocks, which spanning more than 100 genes. In contrast, those between pineapple and Arabidopsis were all located in syntenic blocks that possessed less than 30 orthologous gene pairs. Similar patterns were also observed between pineapple and bananas/maize versus pineapple and grape, which may be related to the phylogenetic relationship between pineapple and other five plant species.

Interestingly, some collinear gene pairs (with seven *AcWRKY* genes) identified between pineapple and rice/maize/bananas were not found between pineapple and Arabidopsis/grape, such as *AcWRKY51/MusaWRKY76*, *AcWRKY51/OsWRKY66*, which may indicate that these orthologous pairs formed after the divergence of dicotyledonous and monocotyledonous plants. Additionally, some collinear pairs (with 14 *AcWRKY* genes) were identified between pineapple and all of the other five species, indicating that these orthologous pairs may already exist before the ancestral divergence.

To better understand the evolutionary constraints acting on WRKY gene family, the Ka/Ks ratios of the WRKY gene pairs were calculated. All segmental and tandem duplicated *AcWRKY* gene pairs, and the majority of orthologous WRKY gene pairs had Ka/Ks < 1, suggesting that the pineapple WRKY gene family might have experienced strong purifying selective pressure during evolution.

### Expression profiling of pineapple *WRKY* genes with RNA-seq

**Fig. 7:**
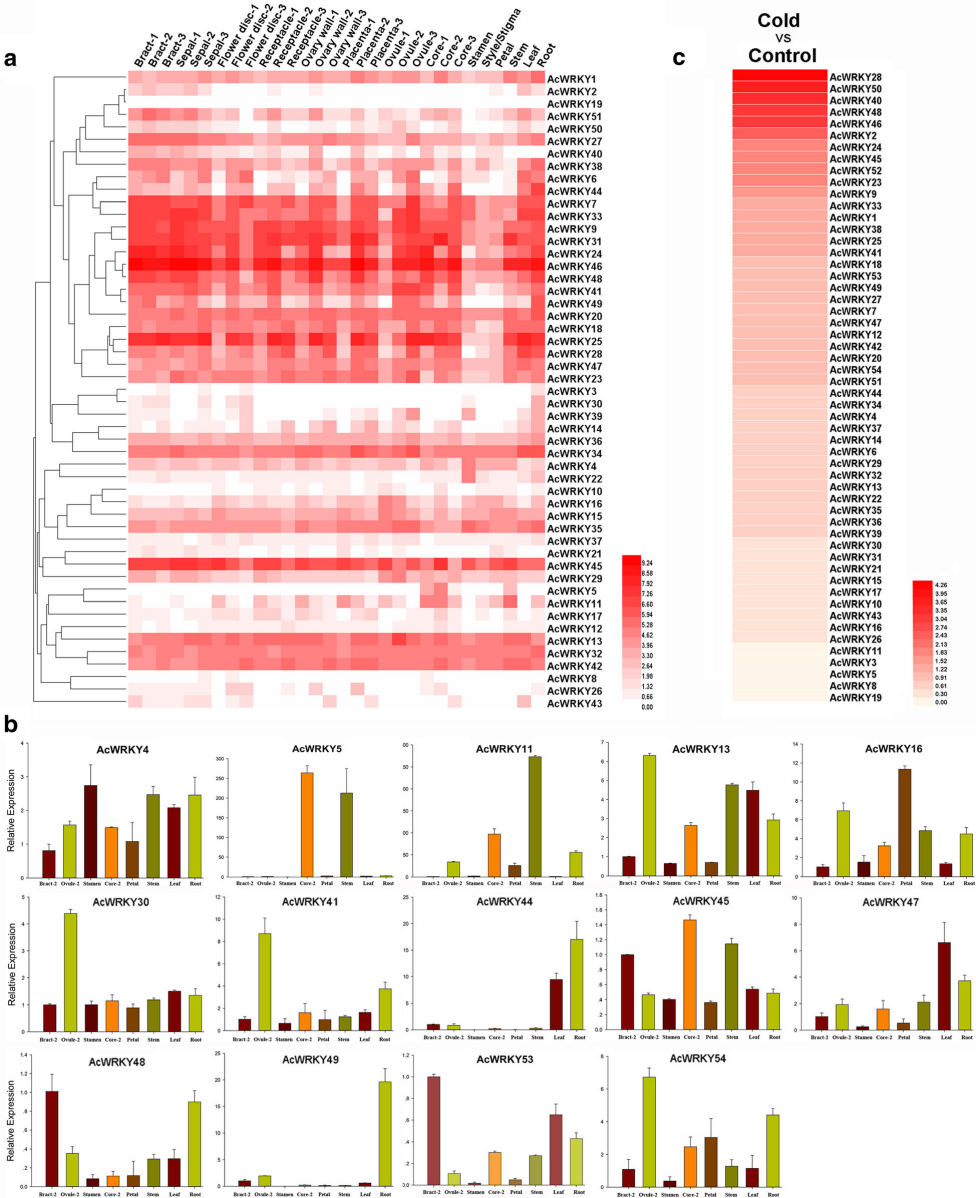
Expression profiles of the pineapple *WRKY* genes. **a** Hierachical clustering of expression profiles of pineapple WRKY genes in 30 samples including different tissues and developmental stages. **b** Expression analysis of 14 WRKY genes in eight representative samples by qRT-PCR. Data were normalized to *β-actin* gene and vertical bars indicate standard deviation. **c** The heatmap exhibit the ratio of the expression levels of pineapple WRKY genes between cold stress treatment and control condition

The expression patterns of all 54 *AcWRKY* genes in the transcriptome data, which was derived from different developmental stages of pineapple organs/tissues, were investigated in this study (Fig. 7a and Additional file 6). The reliability of the transcriptome data was further validated by quantitative real-time PCR (qRT-PCR) experiments which were carried out on eight representative samples for 14 selected WRKY genes (Fig. 7b). Among the 54 *AcWRKY* genes, *AcWRKY19* was not expressed in all detected samples, which may be pseudogenes or had special temporal and spatial expression patterns not examined in our libraries. Thirty-seven WRKY genes were expressed in all 30 samples tested (FPKM > 0) and 28 genes showed constitutive expression (FPKM > 1 in all samples). Some genes exhibited preferential expression across the detected tissues. Eleven genes in root, one gene in stem (*AcWRKY11*), three genes in leaf (*AcWRKY18/25/28*) and two genes in stamen (*AcWRKY4/22*) showed the highest transcript abundances. The expression of some genes exhibited significant trends in different development stages. For example, the expression levels of AcWRKY4 and *AcWRKY25* were gradually reduced along with the fruit core development. The transcripts of *AcWRKY25/31/49* were gradually increased during different developmental stages of ovules.

The transcriptional levels of all 54 *AcWRKY* genes in different whole-fruit developmental stages were also investigated (transcriptome data from Ming et al. [29]), and the results showed that the expression of several WRKY genes were associated with the fruit development (Additional file 7), such as AcWRKY4/13 that with gradually decreased expression patterns. The diel expression analysis of the *AcWRKY* genes in pineapple photosynthetic (green tip) leaf tissues exhibited that three genes (*AcWRKY50/2/53*) had higher expression in dark compared to that in light period (Additional file 7). In case of expression profiles in cold stress library [37], significantly higher expression (Fold change > 2) of 11 *AcWRKY* genes (*AcWRKY28/50/40/48/46/2/24/45/52/23/9*) was observed in cold-stressed samples as compared to control (Fig. 7c).

### Expression patterns of pineapple *WRKY* genes in response to different treatments

**Fig. 8:**
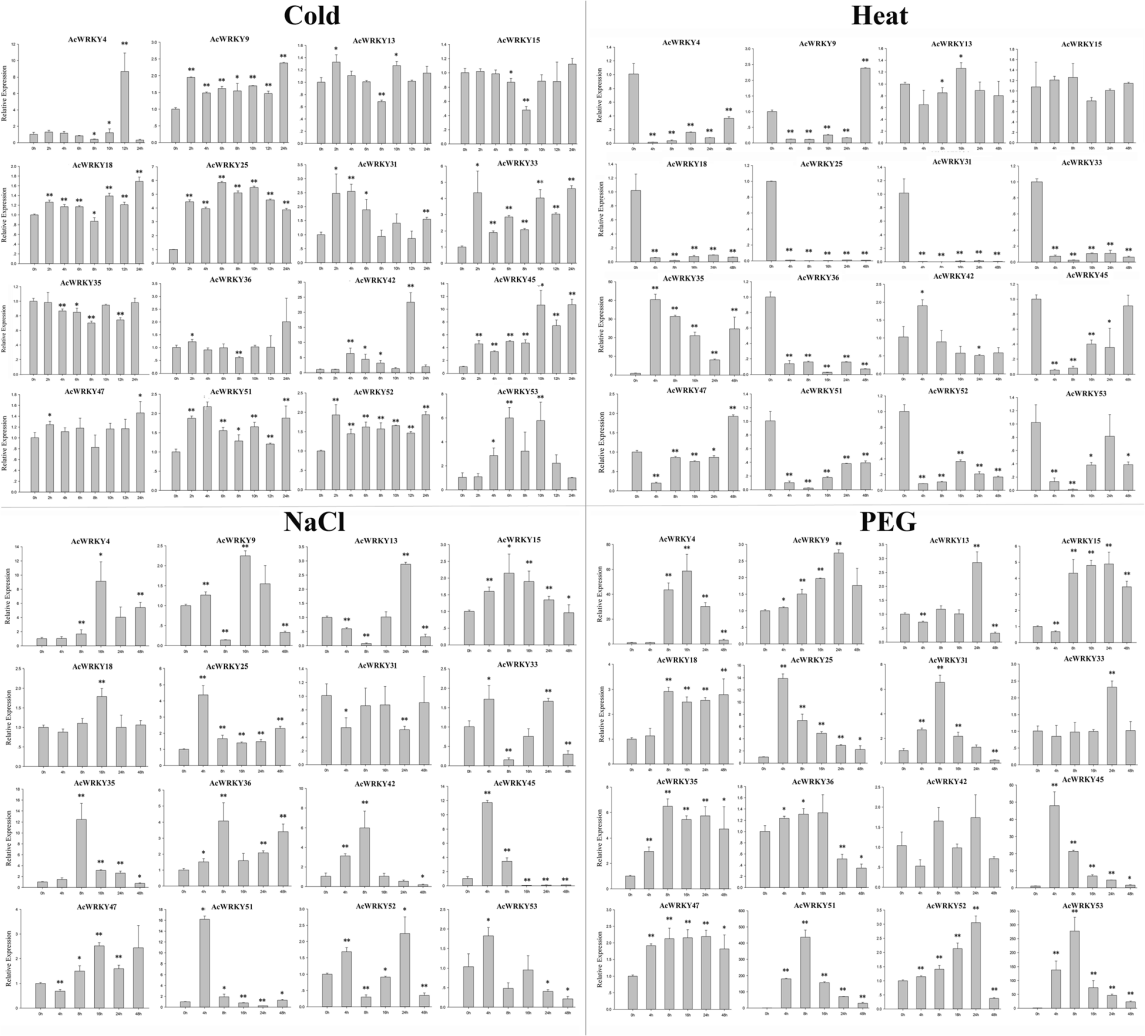
Expression profiles of 16 selected *AcWRKY* genes in response to various abiotic stress treatments. Data were normalized to *β-actin* gene and vertical bars indicate standard deviation. Asterisks indicate the corresponding gene significantly up- or down-regulated compared with the untreated control (*P < 0.05, **P < 0.01, Student’s *t*-test)

**Fig. 9:**
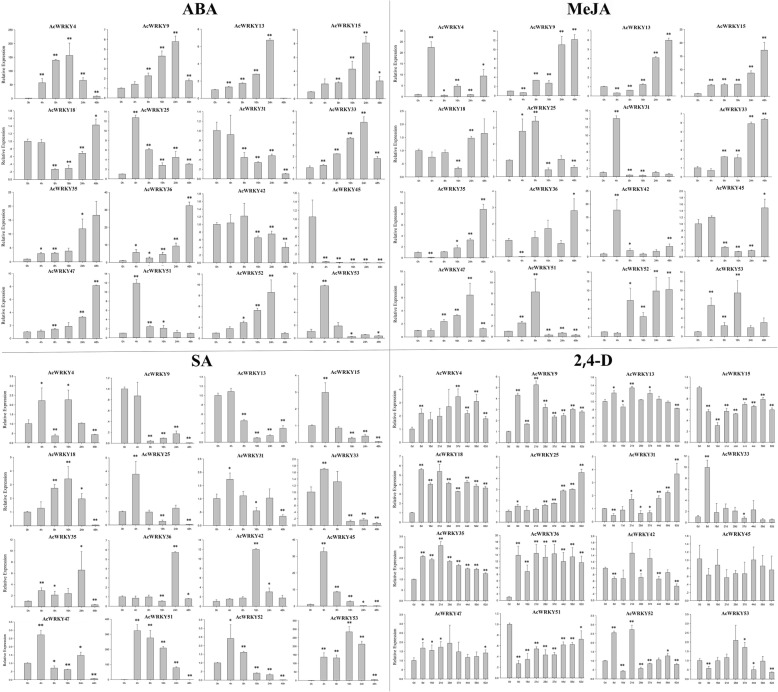
Expression profiles of 16 selected *AcWRKY* genes in response to different hormonal treatments. Data were normalized to *β-actin* gene and vertical bars indicate standard deviation. Asterisks indicate the corresponding gene significantly up- or down-regulated compared with the untreated control (*P < 0.05, **P < 0.01, Student’s *t*-test)

To further confirm whether the expression of *AcWRKY* genes was influenced by different abiotic stresses and hormonal treatments, 16 AcWRKY members, whose mRNA levels were relatively high across different tissues, were carefully selected from 54 pineapple *WRKY* genes. QRT-PCR experiments were further performed to analyze their expression patterns in response to different treatments (Figs. 8 and 9). Overall, some *AcWRKY* genes were significantly induced/repressed by multiple treatments. For instance, *AcWRKY18* significantly responded to SA (Salicylic acid), 2, 4-D (2, 4-Dichlorophenoxyacetic acid), cold and PEG (Polyethylene Glycol) treatments. *AcWRKY35* was induced by all tested treatments except cold stress. In contrast, multiple *AcWRKY* genes were simultaneously induced by one treatment. For example, seven *AcWRKY* genes (*AcWRKY9/18/25/32/45/51/52*) were induced by cold treatment, and five genes (*AcWRKY9/18/35/36/52*) were induced by 2, 4-D treatment. Interestingly, the transcript levels of many *AcWRKY* genes, such as *AcWRKY35*, *AcWRKY36* and *AcWRKY51*, were down-regulated by heat stress treatment. Several genes showed opposing expression patterns under different treatments. For instance, *AcWRKY13* was significantly induced by ABA (Abscisic Acid) and MeJA (Methyl Jasmonate), whereas was repressed by SA treatment.

## Discussion

WRKY genes comprise a large family of transcription factors that are ubiquitous to all plant species. The genome-wide analysis of WRKY gene families have been widely carried out in many species whose genomes have been sequenced [35, 38–40]. In the current study, a search for WRKY gens in the pineapple genome resulted in the identification of 54 members, which were designated AcWRKY1 through AcWRKY54 on the basis of their chromosomal location.

The conserved structural domains of the pineapple WRKY proteins were assessed in this study. Multiple sequence alignments revealed that three AcWRKY proteins (AcWRKY43, AcWRKY28, and AcWRKY23) in group IIc had sequence variation in their WRKY domain. Most characterized WRKY proteins exhibited binding preference to their cognate *cis*-acting W-box element, with the help of WRKY domain. According to previous studies, variations in the WRKYGQK motif in WRKY domain might influence normal interactions of WRKY genes with downstream target genes, and therefore these three WRKY proteins might be worthy to further investigate their functions and binding specificities [41, 42].

The domain gain and loss is a divergent force for expansion of the WRKY gene family. The loss of WRKY domain seems to be common in many monocotyledons such as rice and maize [3, 32, 43]. However, WRKY proteins from group I in pineapple all have two WRKY domains, and no domain loss events were found, suggesting the different characteristics of this group during pineapple evolution. It is reported that the N-terminal WRKY domains showed weak DNA-binding activity and were more variable during evolution. Consisted with previous studies, the pineapple WRKY trees clustered the N-terminal WRKY domains as a monophyletic subtree (Fig. 2), suggesting that the single WRKY domains of group II and III family members are more closely related to the C-terminal domains of group I than to the N-terminal domains [2, 32].

Comparison of the number of *WRKY* genes in pineapple with other sequenced monocot genomes has shown that pineapple possesses comparatively lesser number of genes [34, 43–45]. Tandem and segmental duplication events have played a critical role in the expansion of WRKY gene family [4]. The whole-genome duplication events are common during angiosperm evolution and usually lead to the expansion of gene families [46]. The σ whole-genome duplication (WGD) event was shared with all Poales, while ρ WGD event is inferred to have occurred after divergence of lineages leading to the grasses and pineapple within the Poales [29]. Therefore, lacking the pan-grass ρ WGD event during pineapple evolution might be a possible reason for the smaller amount of *AcWRKY* genes.

Moreover, the variation in the number of group III WRKY genes was also a potential cause of the diversity of WRKY gene family size [31]. The previous studies described group III WRKY genes as being the most dynamic group with respect to gene family evolution. In this study, eight group III *AcWRKY* genes were fewer than that in most other monocots, consistent with the smaller amount of pineapple WRKY family members. Different from the group III WRKY genes in many other monocots, that in pineapple have not undergone a lineage-specific radiation, which may be caused by different pattern of duplication events [30].

Among the WRKY gene family, group III genes were considered as the most advanced in terms of evolution, and seem to have played an important role in plant adaption and evolution [31]. The phylogenetic analysis of the WRKY III proteins could provide more clues about the evolution history of the WRKY gene family [31]. In our study, the plant WRKY III members from the closer related species were tended to be clustered together. Consistent with several previous studies, both monocots and dicots members were present in many clades, indicating that WRKY III genes diversified before the divergence of monocots and dicots [4, 31, 33]. WRKY III genes formed monocot- and dicot-specific subclades depicting that the WRKY genes have evolved independently after the monocot-dicot split. The pineapple lineage diverged from the lineage leading to grasses early in the history of Poales, and become an outgroup and evolutionary reference for the study of the lineage-specific gene family mobility in grasses [47]. The presence of the grasses-specific clade (Clade 3) indicated that the WRKY III genes may have been expanded independently in grasses after the divergence of pineapple and grasses during the Poales evolution. The lineage-specific expansions of WRKY III members in several grass species such as rice and Brachypodium further support this opinion [30, 43]. Similarly, the different evolutionary characteristics were also observed after the divergence of the eurosids group I and II [48].

There is considerable evidence that WRKY genes play significant roles in regulating plant growth and development, and in conferring tolerance to abiotic stresses including salinity, drought, heat, cold and wounding [6, 7, 49]. The Crassulacean acid metabolism plants possess high water-use efficiency and the CAM photosynthesis also confers tolerance to plants against abiotic stress, particularly to drought stress. The genome-sequenced CAM species, pineapple, which was shared conserved syntenic relationships with several important cereal species (like rice and sorghum), has been regarded as an important crop for studying CAM photosynthesis and abiotic stress tolerance [29]. In view of the importance of pineapple in abiotic stress biology and the key roles of WRKY genes in physiological processes and stress responses, the expression patterns of *AcWRKY* genes were investigated in this study, basing on the available transcriptome data and qRT-PCR analysis in response to different treatments. By combining gene expression, phylogenetic and synteny analysis, new clues to the biological function of pineapple WRKY genes could be inferred through comparison with those function-known WRKY genes from model plants.

Some valuable clues about the functional role of *AcWRKY* genes that involved in specific pineapple physiological process were obtained. For example, *AcWRKY7* exhibited the highest expression in mature ovule tissues, while its orthologs in Arabidopsis, *AtWRKY23*, could mediate the embryo development [16], indicating that *AcWRKY7* may share similar functions in pineapple. *AcWRKY5* was specially expressed in stem and fruit core tissues, and with extremely low expression levels in other detected pineapple tissues. Interestingly, its orthologs in Arabidopsis, *AtWRKY12*, was also expressed in stem pith and cortex, where it regulates the secondary cell wall formation [50]. Accordingly, we inferred that *AcWRKY5* may also participate in secondary cell wall formation in corresponding tissues. The pineapple fruit core was developed from inflorescence axis and its firmness was gradually decreased along with the fruit development [26], thus *AcWRKY5* might affect the edible taste of pineapple fruit. *AcWRKY4* was highly expressed in stamen, and have orthologous relationship with two pollen-specific regulators in Arabidopsis, *AtWRKY2* and *AtWRKY34*, indicating that *AcWRKY4* may have similar roles in the pollen developmental modulation [51]. In pineapple photosynthetic leaf tissues, *AcWRKY2* have relative higher expression in dark compared to that in light period (Additional file 7), while its orthologs in Arabidopsis, *AtWRKY40*, was found to be a repressor of high-light-induced signaling and involved in chloroplast dysfunction [52], indicating that *AcWRKY2* may be also associated with the regulation of the high-light stress.

The functional roles of some *AcWRKY* genes which were associated with the various abiotic stresses were also inferred. Low temperature is one of the major environmental stresses that affect pineapple growth and development, and the cold injury usually lead to decreases in crop yield and quality [26, 53]. According to the cold stress transcriptome data [37], the expressions of 11 *AcWRKY* genes were significantly induced. The expression patterns of *AcWRKY9* and *AcWRKY52* were further verified by qRT-PCR analysis (Fig. 8), and they also have phylogenetically closest relationship with *AtWRKY33*, a typical cold-responsive WRKY gene in Arabidopsis, implying their possible roles in pineapple cold stress regulation [54]. The expression of *OsWRKY76* was induced by low temperature, overexpression of *OsWRKY76* in rice plants improved tolerance to cold stress [55], and its orthologs in pineapple, *AcWRKY2*, was also induced by cold stress, suggesting the potential value of *AcWRKY2* in pineapple cold-resistance improvement. Conserved cold-responsive expression features were also observed in orthologous gene pairs like *AcWRKY46-AtWRKY40/18* and *AcWRKY4-AtWRKY34*, suggesting the functional conservation of WRKY genes during evolution [11, 56]. The outstanding drought tolerance was widely found in many CAM species represented by pineapple, and WRKY transcription factors were important components of the drought-stress regulatory network [7]. The drought stress responsive gene *AcWRKY18* was orthologous to *AtWRKY57*, which conferred drought tolerance in Arabidopsis [57], indicating similar function of *AcWRKY18* in drought stress regulation. Accumulating evidence indicated that WRKY genes were regulated by hormones like SA and ABA, and play crucial roles in the hormone signaling network [6, 11]. *AcWRKY9* and its orthologs in Arabidopsis, *AtWRKY25*, were both induced by salt and ABA treatments, implying their similar roles in conferring salt tolerance and ABA signaling network [58]. Consisted with previous studies in other species [6], our current studies showed that some *AcWRKY* genes were differentially expressed following various abiotic stresses and hormone treatments, highlighting the extensive involvement of WRKY genes in environmental adaptation.

It was noteworthy that the expression patterns were not completely consistent between *AcWRKY* genes and their counterparts. For example, *AcWRKY15* and its orthologs in Arabidopsis, *AtWRKY39*, exhibited opposite expression features in response to heat stress [23]. Divergence of gene expression plays an important role in the preservation of duplicated genes. Several pairs of paralogs have different expression patterns, suggesting that they may paly diverse roles in pineapple development. For instance, *AcWRKY4* was preferentially expressed in stamen and cold-responsive, while its paralogous gene *AcWRKY13* was highly expressed across different tissues and cold-insensitive. However, some WRKY genes and their paralogues, like *AcWRKY35* and *AcWRKY47*, shared similar transcript abundance profiles, suggesting that they may have redundant functions.

Overall, these above findings provide insight into the potential functional roles of pineapple *WRKY* genes. The comprehensive analyses were helpful in selecting candidate WRKY genes for further functional characterization, and for the genetic improvement in the agronomic characters and environmental resistance of pineapple.

## Conclusions

A comprehensive analysis of WRKY gene family in pineapple was carried out in the present study. Fifty four full-length WRKY genes were characterized and further classified into three main groups, with high similar exon-intron structures and motif compositions within the same groups and subgroups. Synteny analysis and phylogenetic comparison of WRKY genes from several different plant species provided valuable clues about the evolutionary characteristics of pineapple WRKY genes. *AcWRKY* genes played important roles in pineapple growth and development as indicated by their expression patterns in different tissues and in response to various treatments. The phylogenetic and gene expression analysis will shed light on the functional analysis of *AcWRKY* genes. These results provide a valuable resource for better understanding the biological roles of individual WRKY genes in pineapple.

## Methods

### Gene identification

The hidden Markov model (HMM) file corresponding to the WRKY domain (PF03106) was downloaded from Pfam protein family database (http://pfam.sanger.ac.uk/). HMMER 3.0 was used to search the WRKY genes from pineapple genome database. The default parameters were adopted and the cutoff value was set to 0.01. All candidate genes that may contain WRKY domain based on HMMER results were further examined by confirming the existence of the WRKY core sequences using the PFAM and SMART program. Each potential gene was then manually examined to ensure the conserved heptapetide sequence at the N-terminal region of the predicted WRKY domain. To further check the annotation of the predicted WRKY gene models, available pineapple RNA-seq reads were mapped back to the pineapple genome assembly and gene models, using Tophat2 and Cufflinks [59, 60]. The incorrectly predicted genes were then manually curated and some of them were further validated by PCR amplification and sequencing. The redundant sequences were manually discarded. Fifty-four WRKY gene models were finally identified in the pineapple genome after comprehensive curation. Length of sequences, molecular weights, isoelectric points and subcellular location predication of identified WRKY proteins were obtained by using tools from ExPasy website (http://web.expasy.org/protparam/).

### Sequence analysis

The WRKY domain sequences of the characterized WRKY proteins were used to create multiple protein sequence alignments using ClustalW with default parameters. The deduced amino acid sequences in WRKY domains were then adjusted manually using GeneDoc software. The exon-intron organization of pineapple WRKY genes was determined by comparing predicted coding sequences with their corresponding full-length sequences using the online program Gene Structure Display Server (GSDS: http://gsds.cbi.pku.edu.cn) [61]. The MEME online program (http://meme.nbcr.net/meme/intro.html) for protein sequence analysis was used to identify conserved motifs in the identified pineapple WRKY proteins [62]. The optimized parameters were employed as the following: the number of repetitions, any; the maximum number of motifs, 20; and the optimum width of each motif, between 6 and 100 residues.

### Chromosomal distribution and gene duplication

All *AcWRKY* genes were mapped to pineapple chromosomes based on physical location information from the database of pineapple genome using Circos [63]. Multiple Collinearity Scan toolkit (MCScanX) was adopted to analyze the gene duplication events, with the default parameters [64]. To exhibit the synteny relationship of the orthologous *WRKY* genes obtained from pineapple and other selected species, the syntenic analysis maps were constructed using the Dual Systeny Plotter software (https://github.com/CJ-Chen/TBtools) written by ourselves [65]. Non-synonymous (ka) and synonymous (ks) substitution of each duplicated WRKY genes were calculated using KaKs_Calculator 2.0 [66].

### Phylogenetic analysis and classification of pineapple WRKY gene family

All identified pineapple WRKY genes were divided into different group according to the AtWRKY classification scheme and the alignment WRKY domains of AcWRKY and AtWRKY proteins. The phylogenetic trees were inferred using Neighbor-Joining (NJ) method of MEGA 5.0, with the following parameters: Poisson model, pairwise deletion, and 1000 bootstrap replications. Sequence of WRKY proteins from Arabidopsis, maize [32], rice [43, 67], grape [39], banana [33], and Brachypodium [44] were obtained based on the description in corresponding literatures and downloaded from the phytozome database (https://phytozome.jgi.doe.gov/).

### Plant materials and treatments

*Ananas comosus* cv. Shenwan, a typical cultivated variety, was used throughout the study. The bract, sepal, flower disc, receptacle, ovary walls, placenta, ovule, fruit core from three different developmental stages of pineapple fruit, the stamen, style, petal, stem, leaf and root of mature pineapple plants, were collected separately for RNA extraction and used for further RNA-seq and qRT-PCR analysis. To investigate the expression pattern in response to various stress and hormonal treatments, several *AcWRKY* genes were selected for further qRT-PCR analysis. For salinity and drought treatments, the callus tissues were subjected to a 150 mM NaCl and 15% PEG6000 solution, respectively, for 4, 8, 12, 24 and 48 h. For phytohormone analysis, the calluses were respectively cultured in MS liquid medium supplied with 100 μM ABA, SA and MeJA for 4, 8, 12, 24 and 48 h. For 2, 4-D treatments, the calluses were transferred into the MS solid medium with 4 mg/L 2, 4-D. Samples were collected at 8, 16, 21, 28, 37, 44, 56 and 62 days after treatments. For heat and cold stress treatments, the pineapple plantlets were subjected to 40 and 4 °C, respectively. The leaves were collected at 2, 4, 6, 8, 10, 12, 24 h in cold treatment and 4, 8, 12, 24, 48 h in heat treatment. All treated tissue samples were immediately frozen in liquid nitrogen and stored at − 80 °C for subsequent analysis.

### RNA extraction and gene expression analysis

Total RNA was extracted using Trizol method as described by Ma et al. [68]. All RNA was analyzed by agrose gel electrophoresis and then quantified with a Nanodrop ND-1000 spectrophotometer. DNA-free RNA was used for synthesis of first strand of cDNA by using HiScript® II 1st Strand cDNA Synthesis Kit (Vazyme) as per manufacturer’s recommendations. The quantitative RT-PCR was carried out with the Roche Lightcyler® 480 instrument using SYBR Green chemistry. The housekeeping pineapple *β-actin* gene was used as an internal control. The reaction was carried out as follows: 95°Cfor 30s, followed by 40 cycles of 95 °C, /10 s, 60 °C, /30 s. Each reaction was performed in biological triplicates and the data from real-time PCR amplification was analyzed using 2^−△△CT^ method. Sequences of the primers used in this study were shown in detail in Additional file 8. Details about the transcriptome data derived from various pineapple tissues were described in Liu et al. [64], and the transcriptome analysis of pineapple under cold stress have been carried out in Chen et al. [37]. The transcript abundance of pineapple *WRKY* genes was calculated as fragments per kilobase of exon model per million mapped reads (FPKM). The heatmaps were created by HemI1.0 based on the transformed data of log_2_ (FPKM+ 1) values [69]. The transcriptome data used in this study could also be obtained on the website constructed by ourselves (http://118.24.17.128/html/Pineapple_Expression_DB_By_HeLab_AT_SCAU).

## Additional files

Additional file 1: List of the 54 *AcWRKY* genes identified in this study. (XLSX 60 kb)

Additional file 2: Phylogenetic and HMMER analyses of the R protein-WRKY families. Leaf panel: Neighbor-Joining phylogenetic tree derived from the alignment of full length R protein-WRKY family members. Numbers indicate bootstrap values from 1000 replicates. Right panel: the HMMER-derived overview of protein architecture with predicted protein domains. The length of protein can be estimated using the scale at the bottom. (TIF 279 kb)

Additional file 3: Analysis and distribution of conserved motifs in pineapple WRKY proteins. (XLSX 11 kb)

Additional file 4: Segmentally and tandemly duplicated *AcWRKY* gene pairs. (XLSX 10 kb)

Additional file 5: One-to-one orthologous relationships between pineapple and other five plant species. (XLSX 46 kb)

Additional file 6: RNA-seq data of 54 *AcWRKY* genes that were used in this study. (XLSX 90 kb)

Additional file 7: Expression profiles of pineapple *WRKY* genes in different samples. (A) Expression profiles of pineapple *WRKY* genes in the RNA-seq data derived from different whole-fruit developmental stages. (B) Expression profiles of pineapple *WRKY* genes in the RNA-seq data derived from the pineapple green tip leaf tissues at 2-h intervals over a 24-h period. (TIF 2603 kb)

Additional file 8: Sequences of the primers used in this study. (XLSX 11 kb)

## Abbreviations

2, 4-D: 2, 4-Dichlorophenoxyacetic acid
ABA: Abscisic Acid
AcWRKY: Pineapple (*Ananas comosus*) WRKY
AtWRKY: *Arabidopsis thaliana* WRKY
MeJA: Methyl Jasmonate
PEG: Polyethylene Glycol
SA: Salicylic acid
