# Supplementary figures and images for: Genome-wide investigation of WRKY gene family in pineapple: evolution and expression profiles during development and stress
Source: BMC Genomics. 2018 Jun 25;19:490. doi: 10.1186/s12864-018-4880-x (PMC6019807; doi:10.1186/s12864-018-4880-x)

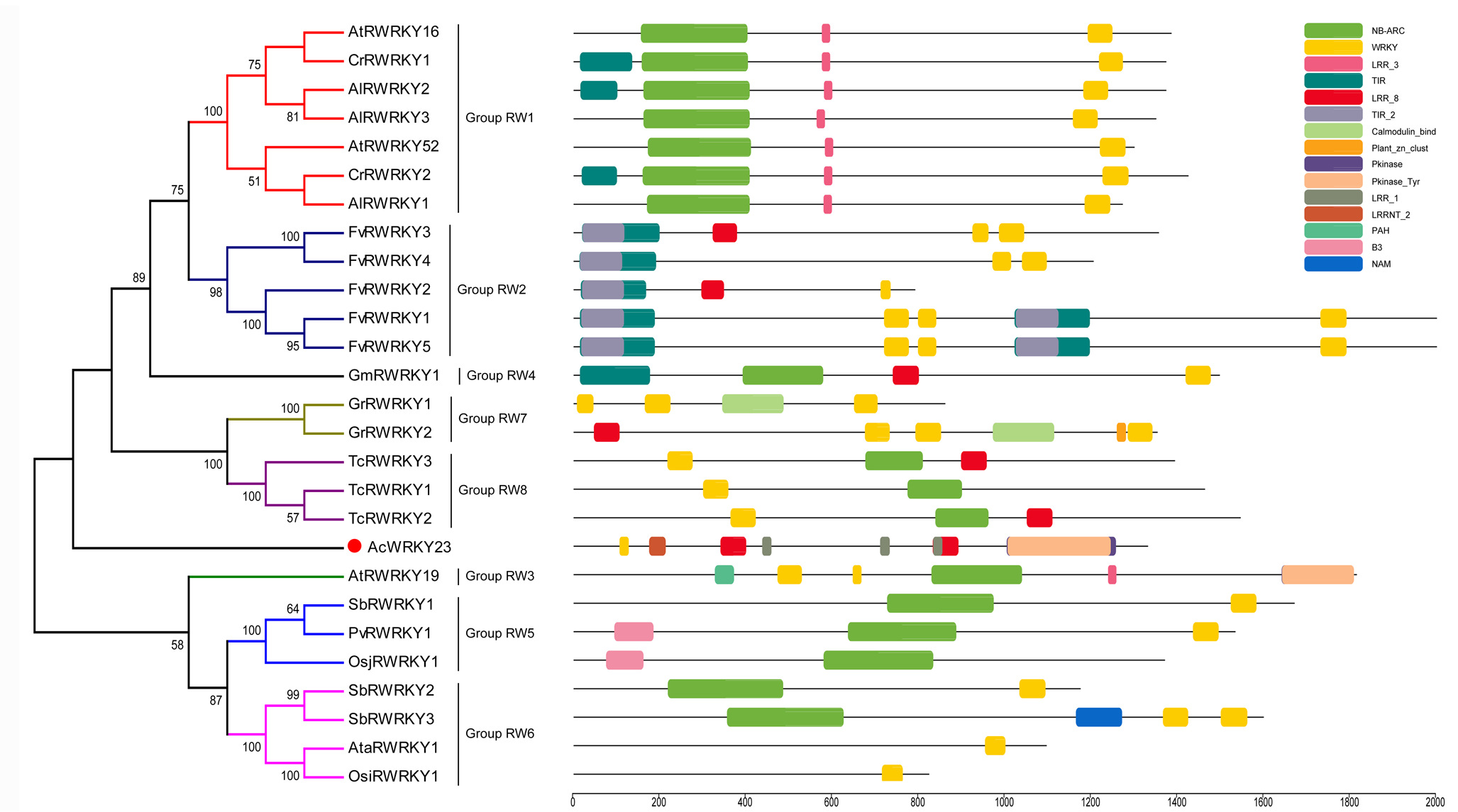

Supplement: Supplementary file 2 — Phylogenetic and HMMER analyses of the R protein-WRKY families. Leaf panel: Neighbor-Joining phylogenetic tree derived from the alignment of full length R protein-WRKY family members. Numbers indicate bootstrap values from 1000 replicates. Right panel: the HMMER-derived overview of protein architecture with predicted protein domains. The length of protein can be estimated using the scale at the bottom. (TIF 279 kb) [file 12864_2018_4880_MOESM2_ESM.tif]

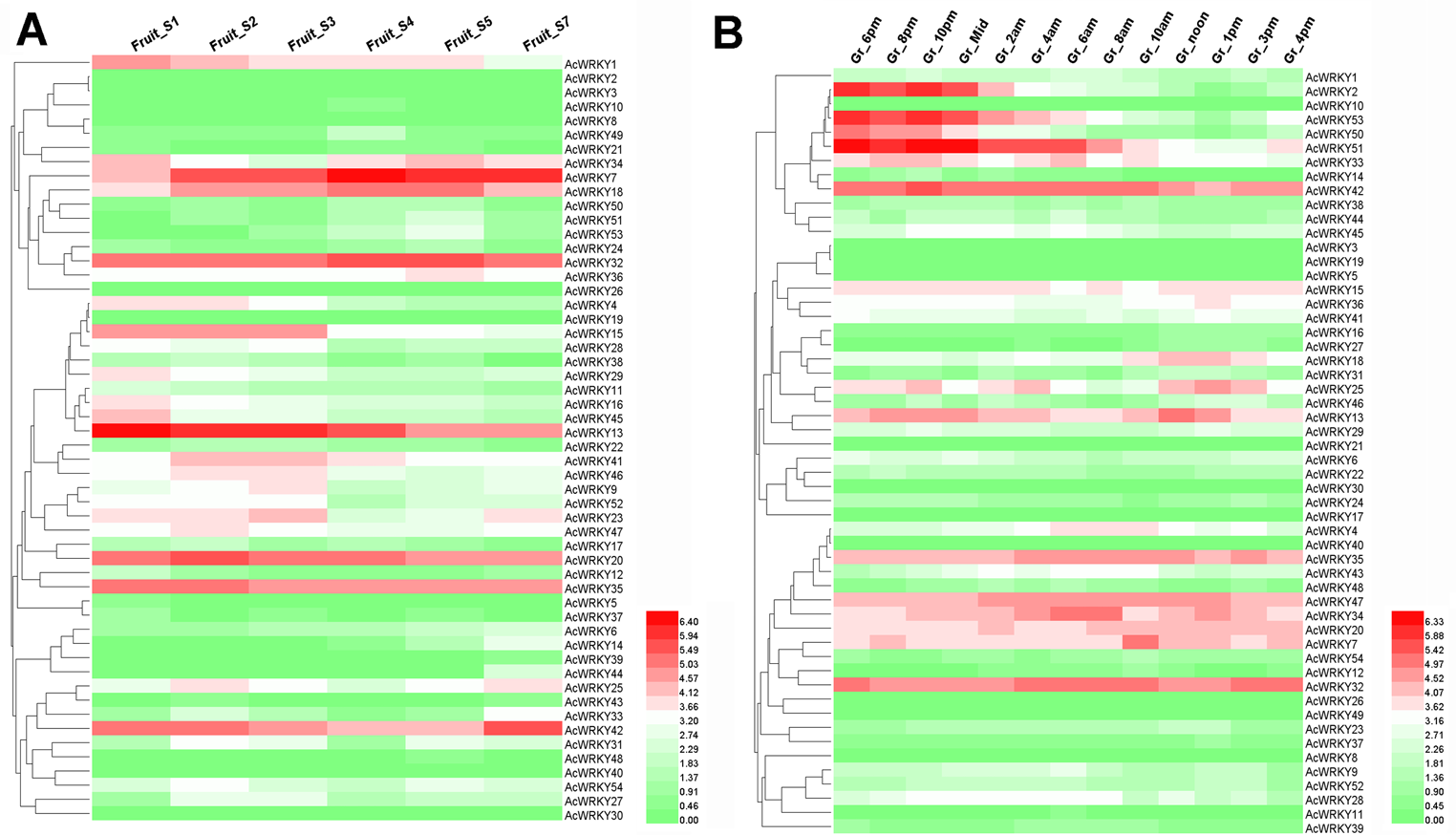

Supplement: Supplementary file 7 — Expression profiles of pineapple WRKY genes in different samples. (A) Expression profiles of pineapple WRKY genes in the RNA-seq data derived from different whole-fruit developmental stages. (B) Expression profiles of pineapple WRKY genes in the RNA-seq data derived from the pineapple green tip leaf tissues at 2-h intervals over a 24-h period. (TIF 2603 kb) [file 12864_2018_4880_MOESM7_ESM.tif]
